# Supplementary material for: The causal role of circulating inflammatory markers in osteoporosis: a bidirectional Mendelian randomized study
Source: Front Immunol. 2024 Jul 18;15:1412298. doi: 10.3389/fimmu.2024.1412298 (PMC11291241; doi:10.3389/fimmu.2024.1412298)
Supplement: Supplementary file 1 [file DataSheet_1.zip › supplementary material/OSTEOPOROSIS-CIM/GCST90274790.tsv.gz_leave_one_out.pdf]

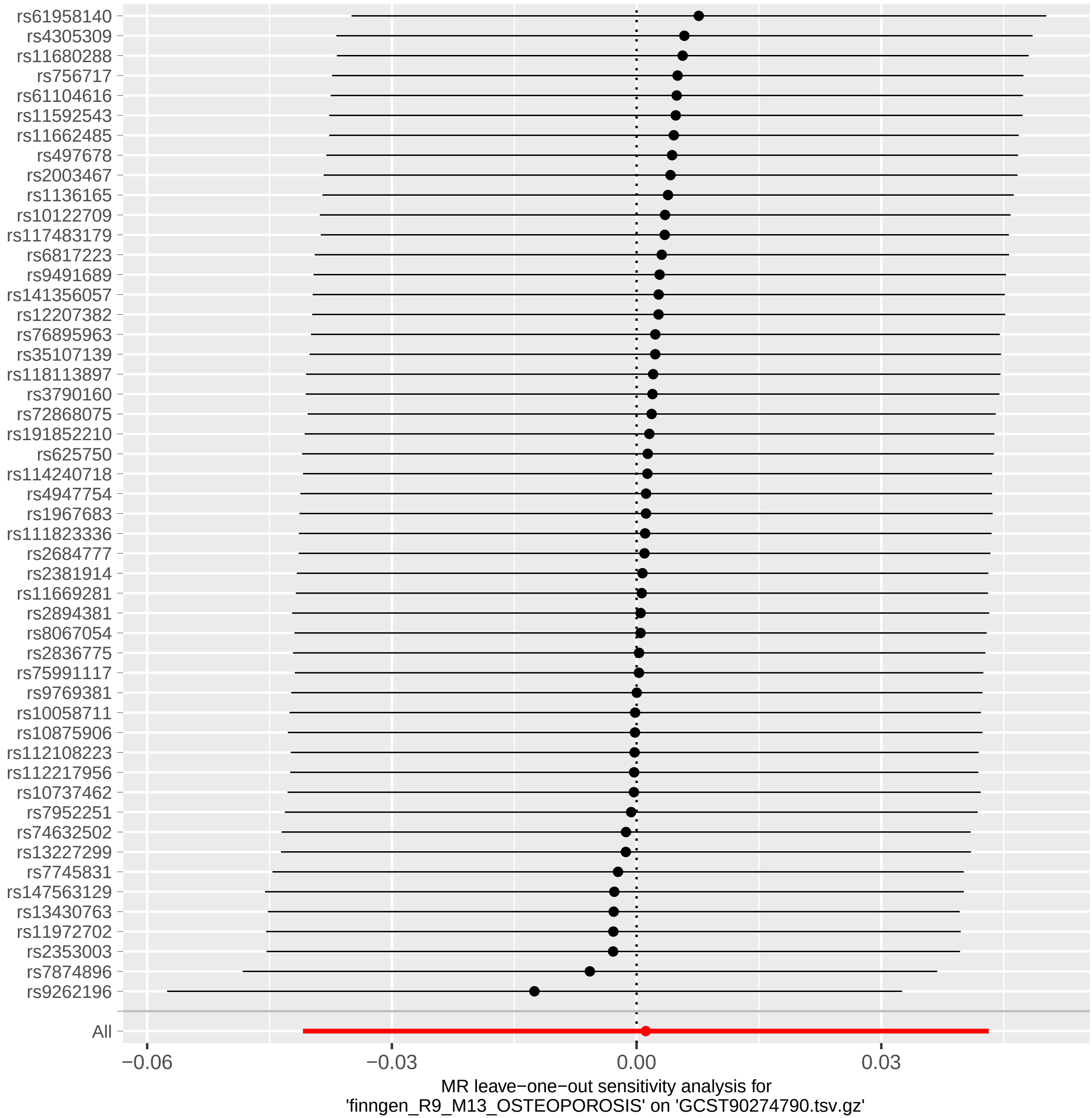
rs61958140
rs4305309
rs11680288
rs756717
rs61104616
rs11592543
rs11662485
rs497678
rs2003467
rs1136165
rs10122709
rs117483179
rs6817223
rs9491689
rs141356057
rs12207382
rs76895963
rs35107139
rs118113897
rs3790160
rs72868075
rs191852210
rs625750
rs114240718
rs4947754
rs1967683
rs111823336
rs2684777
rs2381914
rs11669281
rs2894381
rs8067054
rs2836775
rs75991117
rs9769381
rs10058711
rs10875906
rs112108223
rs112217956
rs10737462
rs7952251
rs74632502
rs13227299
rs7745831
rs147563129
rs13430763
rs11972702
rs2353003
rs7874896
rs9262196
All
−0.06
−0.03
0.00
0.03
MR leave−one−out sensitivity analysis for
'finngen_R9_M13_OSTEOPOROSIS' on 'GCST90274790.tsv.gz'
